# Integrated surveillance systems for antibiotic resistance in a One Health context: a scoping review

**DOI:** 10.1186/s12889-024-19158-6

**Published:** 2024-06-27

**Authors:** Léo Delpy, Chloe Clifford Astbury, Cécile Aenishaenslin, Arne Ruckert, Tarra L. Penney, Mary Wiktorowicz, Mamadou Ciss, Ria Benko, Marion Bordier

**Affiliations:** 1 ASTRE, University of Montpellier, CIRAD, INRAE, Montpellier, France; 2 CIRAD, UMR ASTRE, Dakar, Senegal; 3 National Laboratory for Livestock and Veterinary Research, Senegalese Institute of Research in Agriculture, Dakar, Senegal; 4 Global Food Systems & Policy Research, School of Global Health, York University, Toronto, Canada; 5 Dahdaleh Institute for Global Health Research, York University, Toronto, Canada; 6 Research Group On Epidemiology of Zoonoses and Public Health (GREZOSP), University of Montréal, Saint-Hyacinthe, Québec Canada; 7 Centre de Recherche en Santé Publique de L’Université de Montréal Et du Centre Intégré Universitaire de Santé Et de Services Sociaux (CIUSS) du Centre-Sud-de-L’île-de-Montréal, Montréal, Québec Canada; 8 School of Epidemiology and Public Health, University of Ottawa, Ottawa, Canada; 9 School of Global Health, York University, Toronto, Canada; 10 Institute of Clinical Pharmacy, University of Szeged, Szeged, Hungary

**Keywords:** Antibiotic resistance, Integrated, One Health, Review, Surveillance

## Abstract

**Background:**

Antibiotic resistance (ABR) has emerged as a major threat to health. Properly informed decisions to mitigate this threat require surveillance systems that integrate information on resistant bacteria and antibiotic use in humans, animals, and the environment, in line with the One Health concept. Despite a strong call for the implementation of such integrated surveillance systems, we still lack a comprehensive overview of existing organizational models for integrated surveillance of ABR. To address this gap, we conducted a scoping review to characterize existing integrated surveillance systems for ABR.

**Methods:**

The literature review was conducted using the PRISMA guidelines. The selected integrated surveillance systems were assessed according to 39 variables related to their organization and functioning, the socio-economic and political characteristics of their implementation context, and the levels of integration reached, together with their related outcomes. We conducted two distinct, complementary analyses on the data extracted: a descriptive analysis to summarize the characteristics of the integrated surveillance systems, and a multiple-correspondence analysis (MCA) followed by a hierarchical cluster analysis (HCA) to identify potential typology for surveillance systems.

**Results:**

The literature search identified a total of 1330 records. After the screening phase, 59 references were kept from which 14 integrated surveillance systems were identified. They all operate in high-income countries and vary in terms of integration, both at informational and structural levels. The different systems combine information from a wide range of populations and commodities -in the human, animal and environmental domains, collection points, drug-bacterium pairs, and rely on various diagnostic and surveillance strategies. A variable level of collaboration was found for the governance and/or operation of the surveillance activities. The outcomes of integration are poorly described and evidenced. The 14 surveillance systems can be grouped into four distinct clusters, characterized by integration level in the two dimensions. The level of resources and regulatory framework in place appeared to play a major role in the establishment and organization of integrated surveillance.

**Conclusions:**

This study suggests that operationalization of integrated surveillance for ABR is still not well established at a global scale, especially in low and middle-income countries and that the surveillance scope is not broad enough to obtain a comprehensive understanding of the complex dynamics of ABR to appropriately inform mitigation measures. Further studies are needed to better characterize the various integration models for surveillance with regard to their implementation context and evaluate the outcome of these models.

**Supplementary Information:**

The online version contains supplementary material available at 10.1186/s12889-024-19158-6.

## Background

Antimicrobial resistance (AMR) is a global threat to human health, animal health and the environment. According to O’Neill & al. (2016), AMR could cause 10 million deaths per year by 2050 [1]. These deaths will be mainly concentrated in low- or middle-income countries (LMICs), including 41.5% in Africa and 47.3% in Asia. AMR will also have great impacts on economic growth. The World Bank (2019) estimates that the economic consequences of AMR in 2050 will be more severe than the financial crisis in 2008 [2].

Antibiotic resistance (ABR) plays a critical role in this global crisis. Murray et al. (2019) estimate that ABR was associated with 4.95 million deaths in 2019 [3]. The misuse and overuse of antibiotics in human health, animal health, and food production have resulted in rising levels of ABR [4, 5]. Between 2000 and 2015, global antibiotic consumption increased by 65% [6]. This has contributed to an increase in resistant bacterial strains [1, 7]. ABR surveillance^1^ is crucial to improve knowledge about ABR epidemiology, provide reliable information for evidence-based policy development, and assess the impact of interventions to reduce the threat represented by ABR [8]. In addition, ABR emergence and spread is related to the interactions between the human, animal and environmental sectors [9–11]; its management calls for the development and implementation of strategies in line with the One Health concept [12]. This concept recognizes that the health of humans, domestic and wild animals, plants, and the wider environment are closely linked and inter-dependent, and promotes collaborative efforts across multiple sectors, disciplines and communities at varying levels of society to foster well-being and tackle threats to health and ecosystems [13]. The development of surveillance systems that integrate information about ABR circulating in humans, animals and the environment is critical to enhance our understanding of the complex epidemiology of ABR and to inform policy development and implementation [14].

Since the 1990s, integrated surveillance systems have been widely developed in Europe and North America, including the Canadian Integrated Program for Antimicrobial Resistance Surveillance (CIPARS) in Canada [15], the National Antimicrobial Resistance Monitoring System for Enteric Bacteria (NARMS) in the United States [16], and the Danish Integrated Antimicrobial Resistance Monitoring and Research Programme in Denmark (DANMAP) [17]. There are also some initiatives aimed at compiling national surveillance data at a regional level (e.g. the Joint Interagency Antimicrobial Consumption and Resistance Analysis (JIACRA) programme in Europe [18]) or at the global level (e.g. the Global Antimicrobial Resistance and Use Surveillance System (GLASS) programme [19]). The World Health Assembly in 2015 adopted a Global Action Plan for AMR calling for the development of National Action Plans, which explicitly includes the implementation of systematic, integrated monitoring and surveillance of antimicrobial use (AMU) and AMR [20]. However, despite the existence of National Action Plans in most member states of the World Health Organization (WHO), implementing an integrated surveillance system for AMR and AMU remains a challenge for many countries, in particular in LMICs [21].

There is a growing body of literature describing the development of integrated surveillance for ABR [6, 22, 23]. Several studies examine the establishment of surveillance systems on a regional scale, such as in Asia [24, 25], Africa [26], or Europe [27]. However, to our knowledge there is little published information on the organization and functioning of these existing integrated surveillance systems at a global scale, or on the potential contextual determinants driving their level of integration.

This article intends to fill this gap through a scoping review that analyses: (i) the organizational and functional characteristics of existing integrated surveillance systems for ABR; (ii) the socio-economic and political context in which they operate; and (iii) the levels of integration reached in these systems and their related outcomes. Based on these results, a typology of existing integrated surveillance systems is proposed in order to explore factors that may influence their level of integration.

## Methods

### Definitions

Several definitions have been suggested for integrated surveillance [14, 23, 28] and One Health surveillance [23, 29, 30]. Integrated surveillance conducted with a One Health approach can be defined as a system that applies a collaborative, intersectoral, multi-stakeholder, multi-scale and transdisciplinary approach to improve the functioning and performance of ABR surveillance. In the framework of our study, we use the term integrated surveillance systems to refer to systems that consist of two or more surveillance components implemented in at least two different sectors (of animal health, human health, the environment, and food safety), and that show collaboration at governance and/or operational level. By surveillance component, we refer to a surveillance programme that is supervised by a single institution and implemented by a specific network of actors, and that monitors ABR in one or several populations and/or commodities. By collaboration at the governance level, we refer to any collaborative mechanism (working group, committee, multi-sectoral institution, etc.) across sectors for the steering, coordination or scientific and technical support of the surveillance system. By collaboration at operational level, we refer to any intersectoral modalities for data collection (e.g. harmonized laboratory tests across sectoral surveillance components), data management and storage (e.g. interoperability of databases used in the different surveillance components), data analysis and interpretation (e.g. joint analysis of data collected in the different sectoral surveillance components) and results dissemination and communication (e.g. a joint report including results produced by the different surveillance components).

We recognize that terms AMR and ABR are often conflated, and that AMR is widely used in common language and in the academic literature to refer to ABR; however, we use the term ABR as our research was specifically funded to explore ABR surveillance systems.

### Literature sources and search strategy

The scoping review was conducted according to the PRISMA-ScR guidelines (Preferred Reporting Items for Systematic Review and Meta-Analyses extension for Scoping Reviews) using a systematic search strategy [31, 32]. The literature search focused on primary and secondary peer-reviewed literature in French and English published between 01/01/2000 and 01/08/2022. According to the definition of an integrated surveillance system set for this study, we identified three concepts to be characterized with relevant search terms (See Table 1). Based on these concepts and search terms, we developed strategies to search the following databases: Embase; PubMed; Scopus; and Web of Science. Only title, abstract, and search terms were targeted. The search string was “surveillance” AND “integration” AND “antibiotic resistance”. The complete search terms and index terms used in the search strategy in PubMed are available in Additional file 1 for example and complete replication.

**Table 1:** Terms for search in bibliographic databases

Concepts	Search terms
Surveillance	Surveillance, monitor*
Integration	One health, one medicine, ecohealth, holistic, global health, integrat*, integrat* approach”, integrat* system, integrat* data, inter-sector*, intersector*, cross-sector*, multi-sector*, multisector*, interdisciplinar*, inter-disciplinar*, multidisciplinar*, multi-disciplinar*, trans-disciplinar*, transdisciplinar*, multi-stakeholder*
ABR	Drug resistan*, resistance gene*, AMR epidemiology, microbial sensitivity tests, bacterial resistan*, antibacterial resistan*, antibiotic resistance, antibacterial drug resistance, microbial drug resistance, antibiotic drug resistance, bacterial drug resistance, microbial resistance, antimicrobial resistance, antibiotic drug resistance, bacterial resistance, antibiotics resistance, bacterial, antibiotic susceptibility

^*^truncation operator

### Study selection

All documents retrieved from the bibliographic databases were screened by two reviewers following two distinct steps. For the first step, two inclusion criteria were applied to titles and abstracts: (i) the document describes an integrated surveillance system that meet the definition retained for this study (see the Definitions section); and (ii) the integrated surveillance system includes, at least, a focus on ABR. In the second step, three additional criteria were used: (i) the integrated surveillance system is described in detail, including its organization and operation; (ii) the surveillance system is operational on a continuous basis; and (iii) the surveillance system is operating at a national scale. Bibliographies of selected publications were screened to identify other relevant references. At this step, we also identified grey literature (e.g. reports). In addition, we consulted the websites of international organizations dealing with the ABR issue, e.g., the Food and Agriculture Organization of the United Nations (FAO), WHO, and World Organisation for Animal Health (WOAH, funded as OIE from the French *Office International des Epizooties*), for evidence of existing national integrated surveillance systems.

When documents selected between the two reviewers were different, justification for selection was discussed to make a final decision.

### Data extraction

The process of data extraction from included documents and additional sources is described below. The final list of the 39 variables used to develop the database is presented in Table 2.

**Table 2:** Variables used for the characterization of the integrated surveillance systems for ABR

Domains	Variable groups		Variables	Values
General characteristics	Supervision of the surveillance system	1	Name of the system	Name
		2	Single or multi-institutional lead^a^	Mono; multi
		3	Number of institutions leading the system	Number
		4	Sectors of the institutional lead(s)^a^	Human health; animal health; plant health; wildlife; environment; food safety
		5	Type of institution leading the system	Authority; academia; diagnostic laboratory; non-governmental agency, governmental agency; private practice; private company; technical and scientific organization
		6	Administrative level of the leading institution(s)	Subnational; national
		7	Origin of funds	Domestic; external
		8	Number of surveillance components included^a^	Number
		9	Data owners	∙ Sector (human health; animal health; plant health; environment; food safety) ∙ Type of institution (authorities; academia; diagnostic laboratories; non-governmental agency governmental agency; private practice; private companies; technical and scientific organizations; international organization) ∙ Level (subnational, national)
	System framework	10	Year of establishment of the system^a^	Year
		11	Status of the surveillance system	Stand alone; part of a broader programme
		12	Regulatory status of the surveillance components	Official (owned and implemented by authorities); regulatory (owned by authorities and implemented by other actors); mandatory (owned and implemented by the private sector to meet its regulatory obligations); or voluntary (implemented without any regulatory obligations)
	Geographical area	13	Country where the system operates	Name
	Scope	14	Inclusion of components monitoring antibiotic use (ABU)^a^	Yes; no
		15	Inclusion of components monitoring resistances of other microorganisms^a^	Yes; no
		16	Surveillance strategy	Active surveillance; event-based surveillance
		17	Categories of bacteria under surveillance	Animal pathogens; animal zoonotic bacteria; human pathogens; human zoonotic bacteria; indicator bacteria
		18	Populations/commodities under surveillance^a^	Patients; healthy persons; healthy food animals; diseased food animals; food of animal origin; food of plant origin; healthy companion animals; diseased companion animals; wildlife; water; wastewater; etc
		19	Collection points	Farm; hospital; community; veterinary practice; human medical practice; slaughterhouse; retailer; surface water; wastewater treatment plant; zoo; etc
Objectives and technical performance	Objectives and purposes	20	Objectives of the surveillance system	Early detection; trends
		21	Purposes of the surveillance systems	Rapid response; knowledge improvement; policy development and evaluation; research; risk assessment; awareness; benchmarking; marketing authorization
	Technical performance of the integrated surveillance system	22	Elements supporting evidence of a well-functioning integrated surveillance system	Consistency; continuity; harmonization of laboratory methods among/within sectors; harmonization of interpretative criteria among/within sectors; comparability of data among/within sectors; representativeness of data; system used as a model for other countries
		23	Elements supporting evidence of a suboptimal functioning of the integrated surveillance system	Lack of harmonization of sampling strategies among/within sectors; lack of representativeness of data collected; different type of antibiotics monitored among/within sectors; data quality issue; lack of harmonization of laboratory methods among/within sectors; lack of harmonization of interpretative criteria among/within sectors; lack of comparability of data among/within sectors; multiplicity of components making the coordination difficult; lack of inclusion of the environment
Collaboration for governance	Supervision	24	Sectors involved^a^	Human health; animal health; environment; plant health; food safety
		25	Organizational mechanisms^a^	All components supervised by a single sector; components supervised separately in each sector with participation of other sectors; supervision undertaken by a multi-sectoral body; components supervised separately
		26	Barriers to collaboration for governance	No intersectoral funds; no cross-sectoral coordination mechanisms; no coordination experience; costly coordination
		27	Enablers to collaboration for governance	Existence of cross sectoral mechanisms; few organizations involved in governance
Collaboration for surveillance activities	Data collection and management	28	Organizational modalities^a^	One sectoral organization responsible for all the collection points; one sectoral organization responsible for one or more collection points outside its jurisdiction; each sectoral organization responsible for the collection points under its jurisdiction
		29	Integration point	Harmonization of laboratory methods (within and/or between sectors); harmonization of metrics for reporting (within and/or between sectors); harmonization of data interpretation (within and/or between sectors); interoperable or common information system; one single laboratory for processing all samples; no integration
	Data analysis and interpretation	30	Organizational modalities^a^	All data jointly analysed by one organization; data analysed separately for each component by their supervising organization; data analysed separately for each component by their supervising organization but after harmonization across components; data analysed jointly by all supervising organizations; involvement of the stakeholders in the result interpretation
		31	Integration points^a^	Simple comparison of trends across components; simple statistical correlation; complex statistical modelling; link between ABU and ABR; no integration
	Dissemination to decision-makers and communication	32	Organizational modalities^a^	Information jointly disseminated/ communicated by one sectoral organization for all components; information separately disseminated/ communicated for each component by their supervising organization using separate media; information separately disseminated/ communicated for each component by their supervising organization using a common media; information jointly disseminated/ communicated by all the supervising organizations using a common media
		33	Integration points	Same media for all categories of stakeholders; specific media depending on the categories of stakeholders; no integration
Outcomes	Immediate outcomes	34	Changes in knowledge, awareness, capacities of stakeholders, and in their motivation to collaborate	Capacity to detect correlations in ABR across sectors; capacity to detect correlation between ABU and ABR within sectors; capacity to detect correlation between ABU and ABR across sectors; better understanding of the complexity of ABR; changes in the level of awareness on ABR and ABU
	Intermediate outcomes	35	Changes in practices, behaviours, and interactions	Changes in ABU in humans, animals and in agriculture; changes in prescriber behaviours; integration of a One Health perspective in the development and implementation of sectoral or intersectoral policies in relation to the implementation of the integrated surveillance system; generation of new research programmes on ABR and ABU conducted in a One Health perspective
	Ultimate outcomes	36	Long-term effects (changes resulting from intermediate outcomes)	Changes in ABR levels
Context	Political motivation	37	Country progress with development of a National Action Plan on AMR^a^	No national AMR action plan; national AMR action plan under development; national AMR action plan developed; national AMR action plan being implemented; national AMR action plan being implemented and actively monitored through a monitoring and evaluation framework
	Human density	38	Population density^a^	Number of people per sq. km of land area
	Food animal density	39	Food animals per capita^a^	Number of food animals per capita

*ABR* Antibiotic resistance, *ABU* Antibiotic usage, *AMR* Antimicrobial resistance, *AMU* Antimicrobial usage

^a^Selected variables for the multiple correspondence analysis

#### Data extraction from included documents

To meet the objective of the study, retrieved surveillance systems were characterized against a set of variables that were classified into five categories: (i) general characteristics of the systems (supervision, geographical area, scope, system framework); (ii) objectives and technical performance of the system; (iii) levels of integration for governance (steering, coordination, scientific and technical support); (iv) levels of integration for surveillance operations (data collection and management, data analysis and interpretation, communication and dissemination to decision-makers of the surveillance results); and (v) outcomes of the integration for surveillance (immediate outcomes, intermediate outcomes, ultimate outcomes) (See Table 2). Most of the variables, as well as their possible categories, were based on previous studies evaluating One Health surveillance [14, 33, 34]. However, during data extraction and analysis, some variables were added (e.g., absence or presence of surveillance components monitoring resistance to other antimicrobial agents), revised (e.g., bacteria families changed for bacteria categories depending on their pathogenicity status) or merged (e.g., variables related to steering and coordination merged into a single set of variables related to supervision). Others were dropped because available reliable information was too scarce or subject to a risk of bias (e.g., variables related to the technical and scientific support to the integrated surveillance system, antibiotic classes for which resistance levels were measured, disciplines of people involved in the governance of the systems). We worked extensively on fine-tuning the characterization of the different dimensions of integration that can be found in a surveillance system (see variables describing collaboration for governance and surveillance activities). For some variables (such as the ones related to technical performance, barriers, enablers and outcomes), no values were predetermined and a qualitative content analysis was applied to information available in the database to identify some recurrent themes [35].

If data were missing, additional searches were carried out on the institutional website of the coordinating institutions to retrieve the missing information.

To guarantee the quality of the database, the data extraction was carried out with a double-blind approach. When we identified differences between the two reviewers, differences were resolved by consensus.

#### Data extraction from additional sources

Following the work of Maugeri et al. [36], we included additional information about governance and demography to take into account the implementation context of surveillance. We completed the database obtained with the literature review with data extracted from three other databases: the Global Database for the Tripartite Antimicrobial Resistance (AMR) Country Self- assessment Survey [37] to add information about the countries’ progress regarding development of their respective National Action Plans on AMR, the World Bank Open Data website [38] to add information about human population density, and FAOSTAT [39] to add information about food animal population density.

### Data analysis

We conducted two distinct and complementary analyses of the data collected.

First, we undertook a descriptive analysis of the full database to summarize the characteristics of the integrated surveillance systems retrieved through the scoping review based on the results of the included papers and additional data sources.

Second, we conducted a multiple-correspondence analysis (MCA) using a subset of variables of the full database, followed by a hierarchical cluster analysis (HCA) looking for a potential typology of integrated surveillance systems for ABR. Variables were selected among those for which information could be retrieved for most of the systems and based on their ability to discriminate between these systems and describe their major characteristics (Table 2). A Fisher exact test was applied to variables suspected of being highly correlated (such as “population/commodities under surveillance” and “collection points”). The HCA was applied to the Euclidean distance matrix of the first three axes of the MCA (representing 44% of variance). To check for the robustness of the hierarchical cluster analysis we also conducted a *k*-means clustering algorithm (see Additional file 4).

## Results

**Fig. 1:**
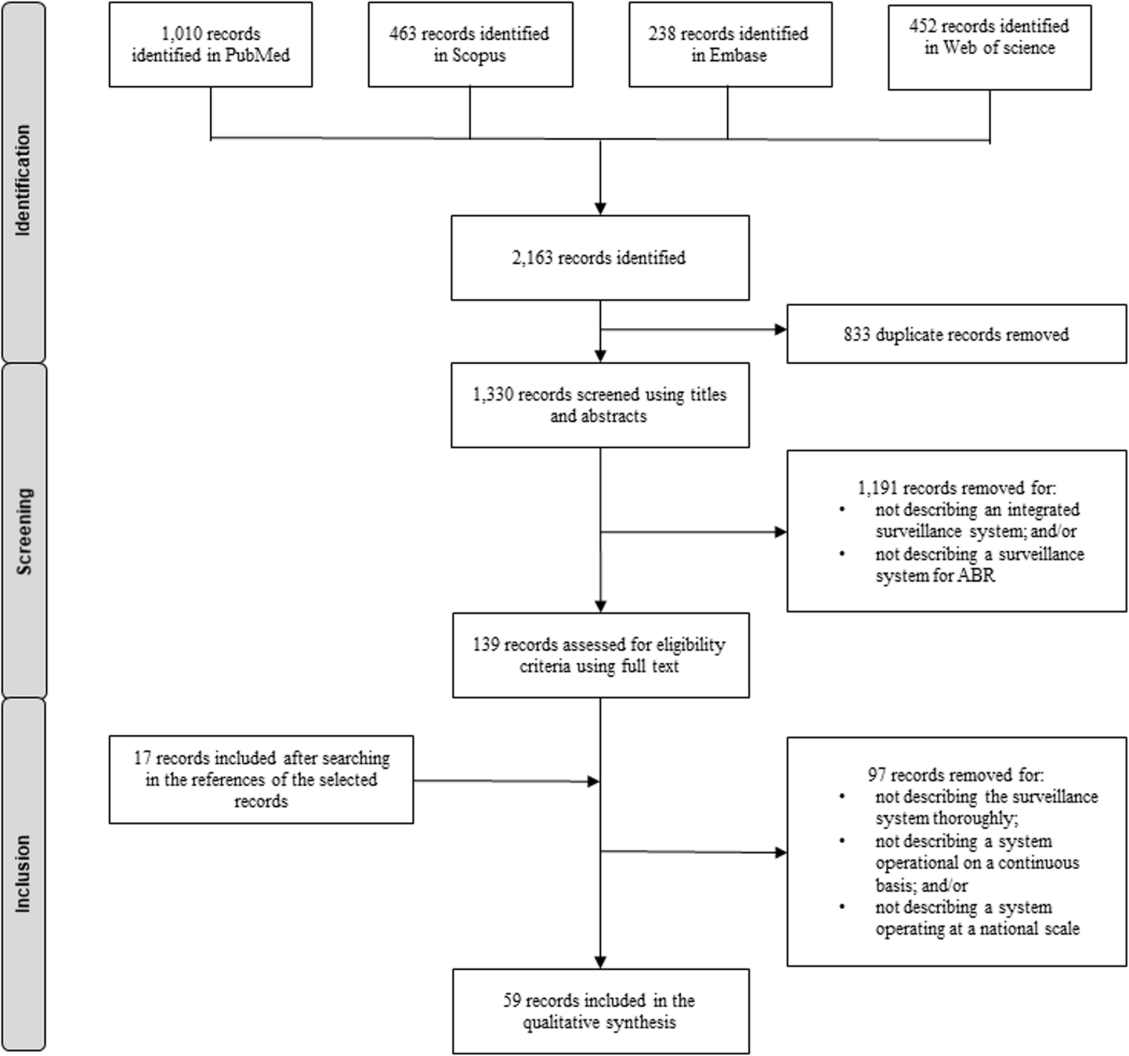
PRISMA-ScR flow chart describing the study selection process for the scoping review

The literature search identified a total of 1,330 records. After the screening phase, 42 references were kept, and 17 additional references retrieved from the bibliographies of the selected references were added (See Fig. 1). From these 59 records, we identified 14 integrated surveillance systems that met our definition (see Table 3). Some systems were excluded from the study because not enough information was available to determine whether they met the inclusion criteria. In particular, systems were excluded for which it was impossible to assess whether they were operational on a continuous basis and not only at a pilot phase [30, 40].

**Table 3:** Main characteristics of the retrieved integrated surveillance systems

Surveillance system, country	Information integration			Structural integration								Typology group	References
	Populations/ commodities under surveillance ^1^	Inclusion of ABU	Inclusion of other AMR	Supervision		Data collection and management		Data analysis and interpretation		Communication and dissemination			
				Organization^1^	Sector^2^	Organization^3^	Integrationpoint ^4^	Organization ^5^	Integration point^6^	Organization^7^	Integration point^8^		
BELMAP, Belgium	Patients Healthy food animals Food of animal origin	X	X	Mono	HH AH	Sectoral	No integration	Sectoral	Separately; link ABU and ABR	Multi sectoral	Same media	**1**	[43, 44]
ONERBA, France	Patients Healthy food animals Diseased food animals Diseased companion animals			Mono	HH F AH	Sectoral	Interoperable information Harmonization for reporting	Sectoral	Separately	Multi sectoral	Same media	**1**	[45–47]
Japan surveillance system, Japan	Patients Healthy persons Healthy food animals Diseased food animals Healthy companion animals Diseased companion animals	X	X	Multi	HH F AH	Sectoral	No integration	Sectoral and harmonized	Comparison of trends; link ABU and ABR; statistical analysis (simple correlation)	Multi sectoral	Same media	**1**	[48, 49]
NethMap/MARAN, Netherlands	Patients Healthy food animals Healthy companion animals Food of animal origin Food of plant origin	X	X	Multi	HH F AH	Sectoral	Single laboratory	Jointly by one	Comparison of trends; link ABU and ABR	Sectoral	Same media	**1**	[50, 51]
ANRESIS / ARCH-Vet, Switzerland	Patients Healthy food animals Diseased food animals Diseased companion animals Food of animal origin Wastewater	X		Multi	HH F AH	Sectoral	Interoperable information	Sectoral	Separately; link ABU andABR	Separately	Same media	**1**	[52, 53]
UK surveillance system, UK	Patients Healthy food animals Food of animal origin	X	X	Multi	HH F AH E	Sectoral	No integration	Sectoral	Separately	Multi sectoral	No integration	**1**	[29, 41]
DANMAP, Denmark	Patients Healthy food animals Diseased food animals Wildlife Food of animal origin	X	X	Multi	HH F AH	Sectoral	Harmonization of laboratory methods Harmonization for reporting	Sectoral and harmonized	Comparison of trends; link ABU and ABR; statistical analysis (simple correlation)	Multi sectoral	Same media	2	[17, 54, 55]
NORM/NORM-VET, Norway	Patients Healthy food animals Diseased food animals Diseased companion animals Wildlife Food of animal origin	X	X	Multi	HH F AH	Sectoral	No integration	Sectoral and harmonized	Comparison of trends; link ABU and ABR	Sectoral	Same media	**2**	[56, 57]
NARMS, United States	Patients Healthy food animals Diseased companion animals Food of animal origin Food of plant origin			Multi	HH F AH	Sectoral	Interoperable information Harmonization of laboratory methods Harmonization for reporting	Jointly by one	Comparison of trends; statistical analysis (simple correlation)	Multi sectoral	Same media	**2**	[16, 58–61]
SWEDRES/SVARM, Sweden	Patients Healthy food animals Diseased food animals Healthy companion animals Diseased companion animals Wildlife Food of animal origin	X		Multi	HH AH	Sectoral	Interoperable information Harmonization for reporting	Multi sectoral	Comparison of trends; link ABU and ABR; statistical analysis (simple correlation)	Multi sectoral	Same media	**2**	[62–64]
CIPARS, Canada	Patients Healthy food animals Diseased food animals Diseased companion animals Food of animal origin	X		Mono	HH	Jointly by one	Interoperable information	Jointly by one	Comparison of trends; statistical analysis (simple correlation, model)	Jointly by one	Same media	**3**	[15, 34, 65–69]
SONAAR, Scotland	Patients Healthy food animals Diseased companion animals	X	X	Mono	HH	Sectoral	Harmonization for reporting	Jointly by one	Comparison of trends; link ABU and ABR	Jointly by one	Same media	**3**	[70, 71]
FINRES-Vet, Finland	Healthy food animals Diseased companion animals Food of animal origin o	X	X	Mono	F	Sectoral	Harmonization of laboratory methods Harmonization for reporting	Sectoral and harmonized	Comparison of trends	Multi sectoral	Same media	**4**	[72]
ZoMo, Germany	Healthy food animals Wildlife Food of animal origin Food of plant origin			Multi	F AH	Sectoral	Single laboratory	Jointly by one	Comparison of trends	Multi sectoral	No integration	**4**	[27, 73, 74]

*1) mono: the system is supervised by a single organization, multi: the system is supervised by several organization;*

*2) HH: human health; F: food safety; AH: animal health; E: environment;*

*3) jointly by one: One sectoral organization responsible for all the collection points; sectoral: each sectoral organization responsible for the collection points under its jurisdiction;*

*4) harmonization of laboratory methods: laboratory methods harmonized across sectors; harmonization for reporting: harmonization of metrics for reporting across sectors; interoperable information: interoperable sectoral information or common information system for the different sectors; single laboratory: one single laboratory in charge of lab testing for all components; no integration: data collection and management conducted independently in each sector without any harmonization;*

*5) jointly by one: all data jointly analysed by one organization; sectoral: data analysed separately for each component by their supervising organization; Sectoral and harmonized: data analysed separately for each component by their supervising organization but after harmonization across components multi sectoral: data analysed jointly by all supervising organizations;*

*6) comparison of trends: simple comparison of trends across components; statistical analysis (simple correlation): simple statistical tools used to analyse correlations of resistance levels across sectors, statistical analysis (model): complex statistical prediction models used to analyse correlations of resistance levels across sectors; link ABU and ABR: investigation of the link between ABU and ABR; no integration: data analysis and interpretation conducted independently in each sector;*

*7) jointly by one: Information jointly disseminated /communicated by one sectoral organization for all components; sectoral: information separately disseminated/ communicated for each component by their supervising organization using separate media; multi sectoral: information jointly disseminated/communicated by all the supervising organizations using a common media;*

*8) same media: same media for all categories of stakeholders; no integration: different media used by the sectors to disseminate and communicate the surveillance results*

### Description of the integrated surveillance systems

#### General description of the integrated surveillance systems

The retrieved systems were found to exclusively operate in high income countries, with domestic funds: 11 in Europe, two in North America, and one in Asia. They were all developed between 1995 and 2018. The surveillance systems include between two and 10 components, with a median of three. These 14 systems operate under different regulatory frameworks: 10 systems include at least one official component (owned and implemented by authorities), 10 at least one voluntary component (implemented without any regulatory obligation), seven at least one mandatory component (owned and implemented by the private sector to meet its regulatory obligations), and six at least one regulatory component (owned by authorities and implemented by other actors). While the stated objective of all the surveillance systems is to follow trends of ABR levels and to detect emergence of new resistances, their final purpose may vary. The most common purposes were developing and evaluating ABR policies, improving the awareness of consumers and health professionals, and improving knowledge and awareness of ABR. More rarely, surveillance systems aim to measure the risks related to ABR and to support the development of research in the ABR field.

The main barrier to the technical performance in these surveillance systems is the lack of harmonization across surveillance components, within and between sectors, in terms of laboratory methods (e.g. NethMap/MARAN, ANRESIS/ARCH-Vet), interpretative criteria (e.g. NethMap/MARAN, ANRESIS/ARCH-Vet, DANMAP), and antibiotic classes monitored (ANRESIS/ARCH-Vet). This leads to a lack of comparability across the data sets that hampers integrated data analysis and interpretation. The lack of harmonization is even more pronounced when there is a high number of surveillance components included, which makes coordination difficult (ONERBA). Articles also point out issues related to the completeness of the data collected, such as the lack of representativity (CIPARS, NethMap/MARAN), and the absence of data collected in the environment (CIPARS). Other hampering factors are more related to the operational organization of the surveillance system, such as the lack of an electronic database or of quality checks of data entry.

The governance of integrated surveillance systems is considered more effective by some authors [17, 34] when an intersectoral committee is in place (DANMAP) or when a single organization is in charge of the supervision of the entire system (CIPARS). Conversely, other studies [29, 41] underlined that intersectoral coordination remains costly and that funding for intersectoral activities are usually lacking, which hampers effective governance of integrated surveillance systems (UK surveillance system). Two systems, CIPARS and DANMAP, are frequently cited as examples of well-functioning surveillance systems, and have served as models for the development of other systems.

Information related to integration outcomes is rarely available in the literature. A few papers describe some immediate outcomes in terms of improvement of: (i) knowledge about ABR (DANMAP, ANRESIS/ARCH-Vet); (ii) surveillance capacity to detect correlation in ABR across sectors (ANRESIS/ARCH-Vet, CIPARS), as well as between ABU and ABR within and across sectors (DANMAP); and (iii) level of awareness on ABR and ABU (Swedres-Svarm). Intermediate outcomes (i.e., changes in behaviours, practices and interactions) were also described. For some systems, integration was found to support prescribers to transition towards a more responsible use of antibiotics (Swedres-Svarm) and to decrease the quantity of antibiotics used in animals and humans (CIPARS, DANMAP, NethMAp/MARAN). New research projects on ABR and ABU conducted with a One Health perspective were also listed among the outcomes of the establishment of integrated surveillance systems (NethMap/MARAN). Finally, in some cases, integrated surveillance has led to the integration of the One Health perspective into the development and implementation of sectoral or intersectoral policies (DANMAP, NARMS). To a lesser extent, we also identified ultimate outcomes of integration (i.e., changes that result from the intermediate outcomes of integration), in terms of changes in ABR levels (CIPARS). As an example, for this chain of outcomes, we can refer to the case of ceftiofur-resistant *Salmonella* in Canada*.* The integrated surveillance system (CIPARS) was able to detect a strong correlation between ceftiofur-resistant *Salmonella enterica* serovar Heidelberg isolated from retail chicken and incidence of ceftiofur-resistant *Salmonella* serovar Heidelberg infections in humans across Canada [42]. As a result, the poultry sector stopped using ceftiofur, which, in turn, triggered a decrease in the level of resistance to this antibiotic in salmonella.

#### Integration levels of the integrated surveillance systems

**Fig. 2:**
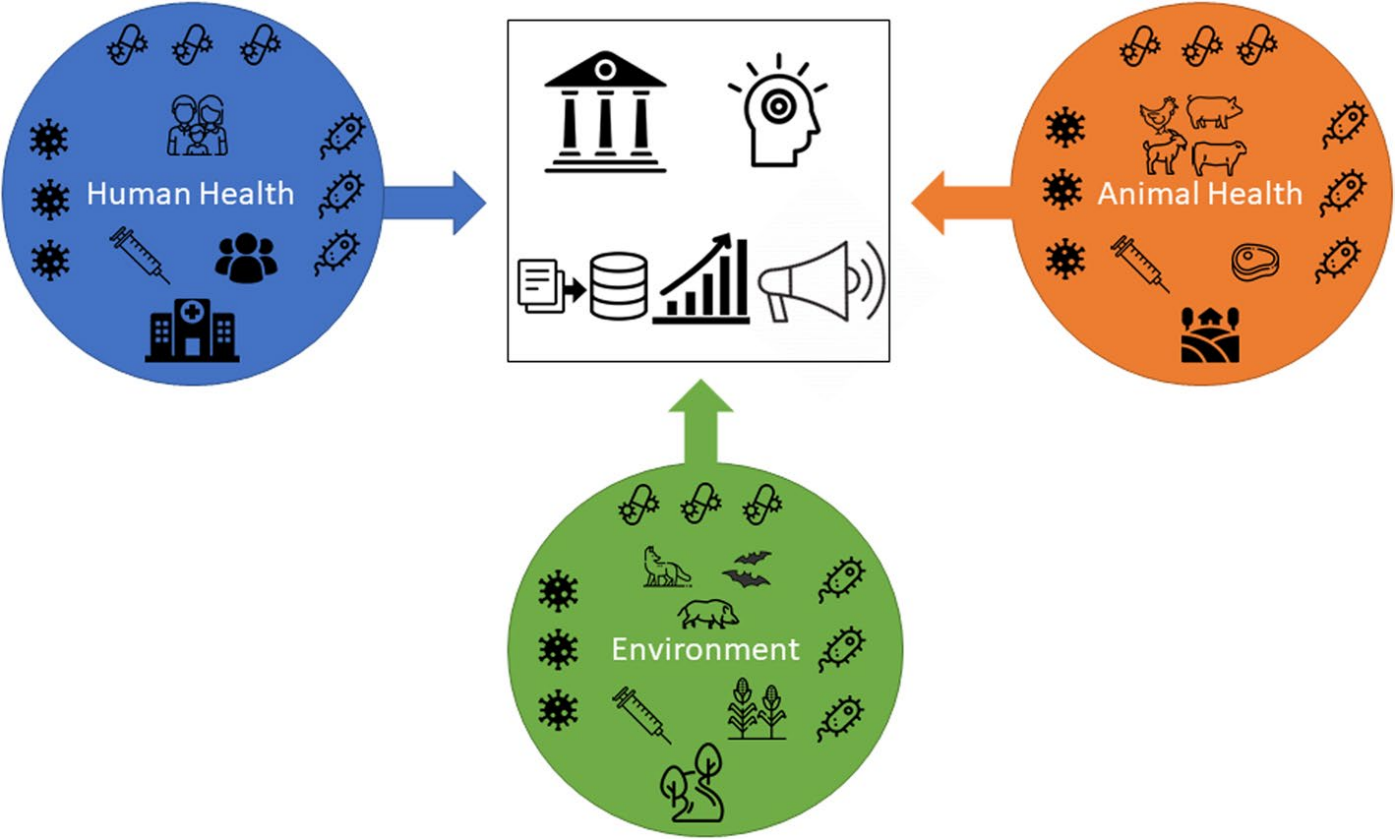
Information and structural integration in surveillance systems for antibiotic resistance. The figure describes the integration in surveillance systems. In the central part, the two structural integration levels are represented: the governance level and the operational level (data collection and management, data analysis and interpretation, communication and dissemination). Each circle represents a sector (human health, animal health, environment) where information may be integrated in terms of populations and commodities, collection points, resistances to other microorganisms, bacteria categories, antibiotic usage and consumption

The analysis of the organization and functioning of the retrieved surveillance systems underlined that integration could be summarized along two dimensions: information integration and structural integration (see Fig. 2).

By information integration, we refer to the ability of the system to combine: (i) data concerning different commodities or populations (human, animal, food, wildlife, water) with different health status (healthy or diseased) and at different collection points (community, health infrastructures, slaughterhouses, waste water management plants, etc.); (ii) data concerning different bacteria family with various pathogenicity characteristics (commensal, species-specific pathogen, zoonotic disease) and resistances to different antibiotic classes; (iii) data generated with different laboratory methods (phenotypic analysis, genotypic analysis) and surveillance strategies (active, event-based); and (iv) data originating from other domains, such as resistances to other microorganisms or usage and consumption data of antibiotics.

By structural integration, we refer to the collaborative mechanisms put in place for the governance and implementation of surveillance activities. At the governance level, collaboration across organizations may exist for the steering of the system (i.e. providing orientations and making decisions for the operations of the system), for its coordination (i.e. ensuring routine operations) and finally for supporting the system technically and scientifically (i.e. providing multidisciplinary support for effective data collection and analysis). At the operational level, collaborative efforts of varying intensity may exist for data collection and management, for data analysis and interpretation, and for the communication and dissemination of surveillance results.

Table 3 displays the descriptive results of the 14 selected systems regarding the variables describing the information and structural integration.

At the level of information integration, the surveillance systems collect samples from three to seven different populations or commodities among diseased humans, healthy humans, healthy food animals, diseased food animals, healthy companion animals, diseased companion animals, foods of animal origin, foods of plant origin, wildlife, and wastewater. For the majority (12 out of 14), they include at least one surveillance component in humans and one in food of animal origin. All surveillance systems collect samples from food animals, mainly at farms and slaughterhouses. Surveillance of human populations at hospital level is part of all systems, except two (ZoMo and FINRES-Vet). Despite the importance of the environment in the emergence and spread of ABR, few systems perform environmental surveillance. Four surveillance systems collect samples from wildlife (mainly in wild boars and foxes) and one system from wastewater (ANRESIS/ARCH-Vet). One system, the FINRES-Vet, also monitors resistances in fur animals, which is consistent with the importance of that sector in Finland. The study identified eight surveillance systems that include the monitoring of ABU. In addition, seven surveillance systems include surveillance components that monitor resistances to other microorganisms, such as fungi or viruses.

At the governance level of the structural integration dimension, five of the surveillance systems are supervised by a single sector, among which four are supervised by the human health sector. For others, the supervision is undertaken either by a multi-sectoral body (four systems) or separately and independently by each sector in charge of the supervision of the surveillance components (five systems). At the operation level of structural integration, all systems except one (CIPARS) demonstrate separate data collection and management operations in the different surveillance components, even if some of them have established some mechanisms to harmonize data collection across components (harmonization of laboratory methods, harmonization of laboratory data interpretation, harmonization of metrics for reporting, interoperable or common information systems, etc.). Integration is further developed during the data analysis and interpretation stage. In only two systems (ONERBA, UK surveillance system) are data separately analysed and interpreted for each of the surveillance components. Among the others, nine compare resistance trends between sectors, five explore resistance correlations between sectors, and eight investigate the link between ABU and ABR. Integration is further developed at dissemination and communication level. Only two surveillance systems (UK surveillance system, ZoMo) have no integration at this stage, while the others publish results using the same media (mainly a written report) intended for all types of audiences (community, decision-maker, etc.).

### Typology of the integrated surveillance systems

The HCA applied to the MCA results demonstrates that the 14 integrated surveillance systems group into four distinct clusters (Table 3 and Additional files 2 and 3). The variables contributing most to variance between individuals (e.g. selected integrated surveillance systems) are: “populations/commodities under surveillance”, “sector involved” in the supervision, “organizational modalities” for data analysis and interpretation, and “organizational modalities” for dissemination to decision-makers and communication. The variable “collection points” was excluded from the MCA because it correlated too closely with the “populations/commodities under surveillance” variable. The values that contribute most to the first axis are: “human health” for the variable “sector involved” in supervision, “human” for the variable “populations/commodities under surveillance”, and “sector involved” for the variable “organizational modalities” for data analysis and interpretation. For axis two, the variable values that contribute most are “jointly by one” for the “communication modalities and “no animal health” and “no food safety” for the variable “sector involved” in supervision. Cluster analysis did not highlight a significant influence of the human and food-animal density on the organization and functioning of integrated surveillance systems, nor of the country progress with development of a National Action Plan on AMR.

Group 1 includes six of the 14 systems: BELMAP (Belgium), ONERBA (France), Japan surveillance system (Japan), NethMap/MARAN (Netherlands), ANRESIS / ARCH-Vet (Switzerland), and the surveillance system in the United Kingdom. In this group, systems consist of at least four surveillance components. Data analysis is carried out separately by the sectoral organizations in charge of the different surveillance components. The system that best represents this group according to the MCA results is the UK surveillance system. The different surveillance components are supervised separately by the leading organization in each sector. Four sectors (human health, animal health, food safety, environment) are involved in the supervision of the system. At the operational level, data analysis and interpretation are carried out separately for each component, and no comparison of ABR occurs among sectors.

Group 2 consists of the following systems: DANMAP (Denmark), NORM/NORM-VET (Norway), SWEDRES/SVARM (Sweden), and NARMS (USA). Unlike the first group, systems are characterized by a relatively limited number of surveillance components (two or three). In addition, except for NARMS, the systems integrate the surveillance of the environment, through the collection of data from wildlife. The DANMAP system best represents this second group. This system is characterized by a high level of information integration (high number of population or commodities under surveillance, inclusion of the surveillance of resistances for other microorganisms than bacteria and of ABU), and by a high level of structural integration at operational level (joint data analysis with comparison of trends across sectors and intersectoral correlation analysis, joint communication and dissemination through awareness campaigns, national reports, and a website). This group therefore differs from the previous one by its high level of integration at both structural and informational levels.

Group 3 includes the Canadian and Scottish systems, respectively CIPARS and SONAAR. These two systems are characterized by leadership of the human sector at governance level. Despite the inclusion of other sectors in surveillance activities (data collection and management, data analysis and interpretation), the human health sector is responsible for supervising these systems. The human health sector is also solely in charge of disseminating and communicating surveillance results. CIPARS best represents this group. It is characterized by strong governance leadership by the human sector. While some CIPARS components target animals and food, they are managed by the Public Health Agency of Canada.

Group 4 includes the German and Finnish surveillance systems, respectively ZoMo and FINRES-Vet. These two systems are supervised by the food safety sector, in collaboration with the animal health sector for the ZoMo surveillance system. Surveillance systems in this group do not collect samples from humans. ZoMo best represents this group. Groups 3 and 4 are opposed in terms of sectoral leadership at governance level. In Group 3, despite the human health leadership, the systems collect data from animals and food. Conversely, the surveillance systems in Group 4 only collect data in domains under their direct supervision (food and animals).

## Discussion

The scoping review retrieved 14 integrated surveillance systems that met the study definition, all operating in high-income countries. They are characterized by various degrees of integration both at information and structural levels. At the information level, they combined surveillance components that collect data about pathogenic or commensal bacteria in a wide range of possible populations and commodities, and some systems expand their coverage beyond ABR. Active surveillance is more frequent in animals, food and the environment, while surveillance in humans mainly relies on resistance data that are collected to orient diagnostic and treatment of patients. At the structural level, the integration modalities differ from one system to another both at governance and operational levels. At one end of the spectrum, we find surveillance systems where supervision is ensured by a single sector, with the consequence that surveillance operation is also highly integrated in terms of data analysis, results dissemination and communication. At the other end of the spectrum, surveillance systems consist of surveillance components that are supervised independently by the sectoral organizations respectively in charge of their operations. Consequently, integration at the operational level is also usually low. The study also found that the selected surveillance systems could be grouped into four distinct clusters. Group 2 is characterized by systems (four) with a high degree of integration, while systems in Group 1 (six) mainly operate in silos with few integration points between surveillance components. Groups 3 and 4 are characterized by the mono-sectoral lead of the systems (except ZoMo). While the two systems in Group 3 are supervised solely by the human health sector, systems in Group 4 (two) are supervised by the food sector, and also by the animal health sector for the ZoMo surveillance system.

### Limits to the information integration in surveillance

Although environmental surveillance is key to understanding the full complexity of ABR and improving its management [75, 76], our review identified very few systems that integrate the environment at both structural (UK surveillance system) and informational (DANMAP, NORM/NORM-VET, SWEDRES, ZoMo) levels. Where the environment is included, this mainly involves wildlife (four systems) or wastewater (one system). There may be several reasons for the lack of inclusion of environment in surveillance. First of all, the environment is a complex system to monitor. The environment is made up of a wide variety of elements (wild animals, plants, water, soil, etc.) and dynamics with different roles in the emergence of ABR. The potential causes of the presence of resistant bacteria in the environment are numerous, and are essentially due to the release of antibiotics from human activities (e.g., agriculture, pharmaceutical industries, hospitals, wastewater management). As a result, it is difficult to establish surveillance protocols, in terms of choice of bacteria, antibiotic families, collection points and sampling strategies. In addition, the environment sector was only introduced in the discussions around the One Health concept at a late stage [77], whereas the animal and human health sectors have a long history of collaboration on health issues [78]. Indeed, while the link between the health of humans and that of animals has been established a long time ago [79], their relation to the health of ecosystems and biodiversity is more difficult to grasp in the world of healthcare [80]. At the international institutional level, this is clearly marked by the integration of United Nations Environment Programme (UNEP) to the Tripartite partnership for One Health [81] in 2022, which was established in 2010 by FAO, WHO and WOAH [82, 83]. Additionally, we did not identify any surveillance systems whose surveillance targets resistances in plant production, even though antibiotics are now used in agriculture to protect crops against pests [84].

Apart from ABU, which is recognised as a key driver of ABR, other potential drivers of ABR emergence and spread were not considered in the retrieved surveillance systems. However, ABR may be influenced more or less directly by a wide range of factors, such as climatic drivers (temperature, precipitations), meat consumption or chemical levels (responsible for potential co-selection of resistances), that could be easily included in integrated surveillance systems for a more comprehensive understanding of ABR epidemiology and a better orientation of interventions to mitigate the risk of ABR [85–87].

While information integration through the harmonization and combination of data from different sources will undoubtably improve the surveillance effectiveness, this should not be done at the expense of the surveillance needs in the respective sectors [88]. Moreover, better integration does not systematically bring greater surveillance performance, as integration is associated with a financial and social cost and may not achieve the expected positive outcomes if the implementation context is unsuitable (e.g. poor quality of data produced by the surveillance components) [21].

### Integrated surveillance in low- and middle-income countries

A country's level of resources seems to play a predominant role in the implementation of integrated surveillance systems. Despite the adoption of many National Action Plans for AMR, the review was not able to identify operating integrated surveillance systems in LMICs [89]. This can be explained by several reasons. First, resources for disease prevention, and epidemiological surveillance in particular, are scarce compared to those for medical care. As a result, sectoral surveillance components are usually low-performing and produce data of a quality that is not suitable for further combination with other sources of data [88]. The literature highlights several technical (information technology systems, expertise, infrastructure), budgetary, and institutional (political instability, weak enforcement of regulations) constraints that hamper the implementation of integrated surveillance systems in LMIC [90, 91]. In Nepal specifically, Malla et al. [92] highlight several gaps in sectoral surveillance performance that compromise the ability to further integrate the information across different domains, such as: the lack of appropriately trained personnel; high turnover of staff; poor access to good quality reagents; inadequate storage facilities of reagents; and frequent power cuts. Secondly, as described previously, integration of information requires a well-established structural integration through the development of mechanism to ensure an inter-sectoral governance of the surveillance. Indeed, only strong governance with coherent provisions can support the operationalization of the One Health concept, including the establishment of integrated surveillance systems [93]. Despite the international effort to support LMICs in establishing intersectoral mechanisms for health management at a high political level, One Health governance is still struggling to become operational in many countries and this hampers the development of integrated surveillance [94]. One Health policy-making is still fragmented and the distribution of roles and responsibilities among ministries for the establishment and operation of integrated surveillance lacks transparency [91, 95]. Additionally, in LMICs, national action plans are generally drawn up under the impetus of international organisations, but rarely receive the required domestic resources to implement them. Finally, despite the alarming figures available on the costs and deaths incurred by resistant bacteria [96, 97], the immediate and visible impacts of ABR on health and economics, as compared to other communicable or non-communicable diseases, are not always clear to stakeholders. As a result, ABR is not among the top priorities for governments who must manage a multitude of health issues on a day-to-day basis with limited resources [88]. This low inclusion of LMICs in the race for ABR surveillance performance is problematic, as they also play a role in the ABR crisis [98, 99]. LMICs usually face high levels of resistance because of, among others, inappropriate prescription practices, inadequate antibiotic user education, limited diagnostic facilities, informal sale of antibiotics, lack of appropriate functioning drug regulatory mechanisms, and use of antibiotics in animal production [99]. Whether through international trade, particularly in the livestock sector [100], or international tourism [101], the spread of ABR is inevitable and therefore requires a global approach to mitigate the risk. As long as integrated surveillance is not generalized on a global scale, a gap will remain in the development of effective policies to curb the development of ABR.

Especially in LMICs, a tiered approach to integration is needed. First, it is essential that each surveillance component included in the integrated surveillance system can produce quality data. Then, a first step could consist of involving relatively standard collection, sharing and analysis of data. In a context of scarce resources, the prioritization of sources to be integrated into ABR surveillance systems could be based on the following criteria: availability of existing samples, that have been collected for other purposes (e.g. samples taken for diagnostic purposes), limited need for resources to carry out the sampling (e.g. samples taken at the abattoir), production of information to guide the implementation of prompt AMR containment measures, socio-economic and health characteristics of the implementation context (e.g. food consumption patterns in the human population and the expected prevalence of ABR in animal populations). Then, the system could progress to higher tiers by broadening data collection sources and by conducting more complex data analysis [14, 22].

### Integrated surveillance and international governance

The institutional and regulatory framework at a supranational level appeared to shape the organization and functioning of integrated surveillance systems. Among the 14 integrated surveillance systems that the scoping review identified, 11 were established in the member countries of the European Union (EU). This can be explained by the strong institutional regulatory framework for ABR surveillance and reporting that member countries must comply with. In addition to the fact that EU countries are required to implement intersectoral national plans in line with EU One Health Antimicrobial Resistance Action Plan to combat ABR [102], they are also requested to have systems in place to monitor ABR in humans and animals, from farm to fork. As a result, surveillance is usually strongly integrated along the food chain for food of animal origin in all countries of the EU, and depending on the countries, with the surveillance components in humans. EU countries are also required to report their AMR and AMU data through the European Antimicrobial Resistance Surveillance Network (EARS-Net) and the European Surveillance of Antimicrobial Consumption (ESAC-Net), supervised by the European Centre for Disease Prevention and Control (ECDC), which is responsible for compiling reports on the AMR and AMU situation at the EU level [18].

While a strong institutional and regulatory framework at regional level seems efficient to favour integrated surveillance, global policies struggle to deliver concrete results at country level. International organizations have issued strategies and guidelines to support the development of integrated systems. In addition to the Global Action Plan on AMR [20], the Codex Alimentarius released guidelines on integrated monitoring and surveillance of foodborne AMR in 2021 [103] and the WHO advisory group a guideline on Integrated Surveillance of Antimicrobial Resistance (AGISAR) in 2017 [104]. As a result, 90% of all countries, and 61% of LMICs, show a National Action Plan for AMR in 2023 [105], and the majority aim to develop integrated surveillance systems for AMR [106]. However, our scoping review did not identify operational integrated surveillance systems in LMICs. In addition to the technical, institutional, political and budgetary constraints mentioned previously, the lack of operationalization of global policies at national level in LMICs can also be explained by the failure to apply the same models in countries with heterogeneous socio-political and economic contexts [107, 108] Because of considerable LMIC reliance on external budgets for their health policies, their national action plan is usually highly influenced by the agendas of the technical and financial partners who support them in the fight against AMR. As a result, countries are usually simply mimicking the Global Action Plan and may propose actions that are irrelevant to the local context and/or to the expectations and constraints of local actors who are the most impacted [94].

### Limitations and perspectives

Our study has several limitations. First, we identified integrated surveillance systems only in high-income countries. We may have missed some more recent systems that are not yet covered in the published literature. Indeed, many institutional reports describing National Action Plans exist that include the establishment of integrated surveillance systems [89]. However, we were not able to verify whether those systems were implemented. In addition, the study failed to deeply characterize some aspects of integration reached in the surveillance systems, as well as the influence of integration on system outcomes. Indeed, information about performance and outcomes are particularly scarce in the literature. When available, the direct causal link with the integration is difficult to establish and information is subject to caution in terms of validity as it is usually provided by people in charge of the surveillance system, who may lack objectivity. Reliable information regarding performance and outcomes could only be found for systems at an advanced stage of maturity that have undertaken external evaluations [22]. This leads to a certain paradox in our results. Weaknesses and gaps were mainly found for the best-performing systems because they are the only ones subject to robust evaluation with the objective of continuous improvement. Less performant and more recent systems are usually not evaluated, and information about performance and positive outcomes gives an impression of being more aspirational than based on observed facts. The fact that few existing systems have been formally evaluated to assess their effectiveness and impacts can be partly explained by the lack of adapted tools and methods to evaluate the added value of integrated surveillance [14, 94]. This study reiterates the need to enhance such methods and to conduct more evaluation of the impacts of integrated surveillance systems in order to better understand the mechanisms through which the integration process enhances surveillance effectiveness and value [21]. The use of mixed methods that integrate both quantitative and qualitative approaches could help address the complexity of the relationship between surveillance outputs and impact on decision-making [34, 109]. This could help identify best practice for integrated surveillance system development by assessing the role that integration characteristics play in the success of surveillance system success – characteristics such as those described in our review, but also those for which data availability was too limited for inclusion (e.g., level of multi-disciplinarity in teams operating surveillance systems).

## Conclusion

This scoping review led to the identification of 14 surveillance systems for ABR that demonstrate a great diversity of collaborative efforts for the governance and/or operation of surveillance activities between at least two distinct surveillance components. They can be grouped into four clusters characterized by the nature of integration at the informational and structural level. The study highlighted the absence of documented integrated surveillance systems in LMICs and the low inclusion of the environmental data and risk factors to ABR emergence and diffusion in surveillance efforts. Regarding the global dimension of the ABR crisis and the crucial role of the environment in the ABR dynamics, effective policies and interventions to reduce ABR levels can only be achieved if all countries embark on the establishment of integrated surveillance systems covering the key three sectors (human health, animal health, environment). Moreover, regarding the diversity of mechanisms driving global ABR, an extension of the surveillance scope beyond ABR and ABU data is needed to provide necessary information to support the development of efficient mitigation measures.

This study explored only partially the link between integration and context on the one hand and integration and outcomes on the other. The typology identified provides a starting point for investigating these two aspects more deeply, using adapted evaluation methods that would include both quantitative and qualitative approaches.

Moving towards an integrated surveillance system for ABR is a stepwise approach that must be tailored to the resource availability and the socio-economic and epidemiological characteristics of each country. Consequently, it is important to define a national roadmap for setting up an integrated surveillance system in a concerted and participatory manner with all local and central actors, in a way that the system is adapted to the broader context, other priorities and the expectations of the stakeholders, and is therefore accepted and sustainably implemented [94]. Our analysis can provide a starting point for governments when reflecting on the right integration set-up for building integrated ABR surveillance systems. But future research in this area needs to develop evaluation tools specific to integrated ABR surveillance in order to better understand how to improve surveillance effectiveness and demonstrate the added value of integration.

### Supplementary Information

Supplementary Material 1.

Supplementary Material 2.

Supplementary Material 3.

Supplementary Material 4.

## Data Availability

The full dataset generated during the current study is available from the corresponding author on reasonable request.

## Footnotes

1 “Surveillance” here is taken to mean the continuous collection of health data in a given population to study changes in health status over time and space, in order to inform decision-making for the reduction of related risks.
