# Supplementary material for: Integrated surveillance systems for antibiotic resistance in a One Health context: a scoping review
Source: BMC Public Health. 2024 Jun 27;24:1717. doi: 10.1186/s12889-024-19158-6 (PMC11210117; doi:10.1186/s12889-024-19158-6)
Supplement: Supplementary file 1 — Supplementary Material 1. [file 12889_2024_19158_MOESM1_ESM.docx]

Additional file 1. search terms and index terms used in the search strategy (PubMed)

| **Terms connected by OR** | **AND** | **Terms connected by OR** | **AND** | **Terms connected by OR** |
| --- | --- | --- | --- | --- |
| "Surveillance"[tiab]  OR "Monitor*"[tiab] |  | "One Health"[tiab]  OR "One medicine"[tiab]  OR "Multistakeholder*"[tiab]  OR "Multi -stakeholder"[tiab]  OR "Integrated"[tiab]  OR "Integrating"[tiab]  OR "Inter-sector*"[tiab]  OR "Intersector*"[tiab]  OR "Cross-sector*"[tiab]  OR "Multi-sector*"[tiab]  OR "Multisector*"[tiab]  OR "Interdisciplinar*"[tiab]  OR "Inter-disciplinar*"[tiab]  OR "Multidisciplinar*"[tiab])  OR "Multi-disciplinar*"[tiab]  OR "Trans-disciplinar*"[tiab]  OR "Ecohealth"[tiab]  OR "Planetary Health"[tiab] |  | "Drug resistan*"[tiab]  OR "Resistan* gene*"[tiab]  OR "AMR epidemiology"[tiab]  OR "Bacterial resistan*"[tiab]  OR "Antibacterial resistan*"[tiab]  OR "Antibiotic resistan*"[tiab]  OR "Antibacterial drug resistan*"[tiab]  OR "Microbial drug resistan*"[tiab]  OR "Antibiotic drug resistan*"[tiab]  OR "Antimicrobial resistan*"[tiab]  OR "Bacterial drug resistan*"[tiab]  OR "Microbial resistan*"[tiab]  OR "antibiotic usage*"[tiab]  OR "antimicrobial usage*"[tiab] |
