# Supplementary figures and images for: Integrated surveillance systems for antibiotic resistance in a One Health context: a scoping review
Source: BMC Public Health. 2024 Jun 27;24:1717. doi: 10.1186/s12889-024-19158-6 (PMC11210117; doi:10.1186/s12889-024-19158-6)

Additional file 2. Individual plan of the MCA


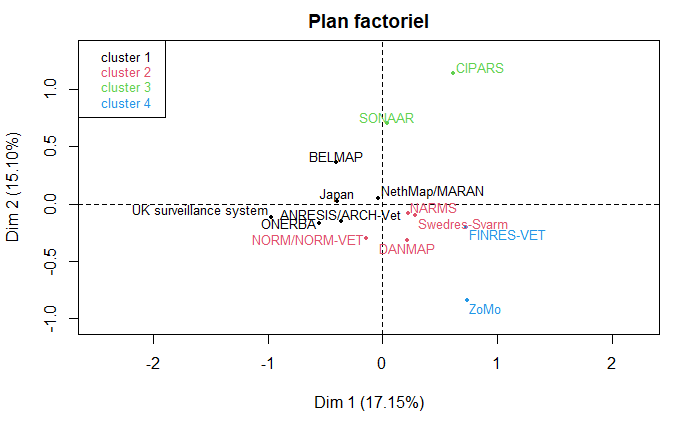

Supplement: Supplementary file 2 — Supplementary Material 2. [file 12889_2024_19158_MOESM2_ESM.docx]

Additional file 4. Clustering with *k*-means method.
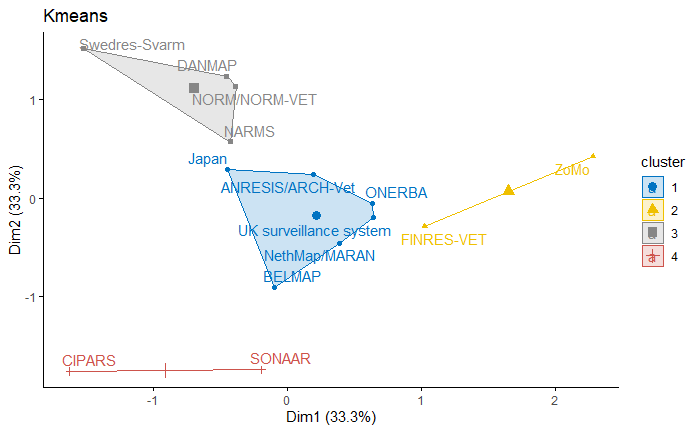

Supplement: Supplementary file 4 — Supplementary Material 4. [file 12889_2024_19158_MOESM4_ESM.docx]
