# Supplementary material for: Integrated surveillance systems for antibiotic resistance in a One Health context: a scoping review
Source: BMC Public Health. 2024 Jun 27;24:1717. doi: 10.1186/s12889-024-19158-6 (PMC11210117; doi:10.1186/s12889-024-19158-6)

Additional file 3. Dendrogram of existing integrated surveillance systems using hierarchical cluster analysis.


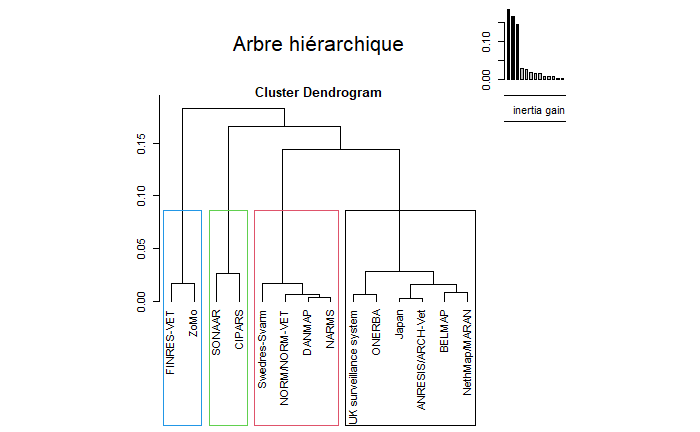

Supplement: Supplementary file 3 — Supplementary Material 3. [file 12889_2024_19158_MOESM3_ESM.docx]
